# Estrogenic chemicals often leach from BPA-free plastic products that are replacements for BPA-containing polycarbonate products

**DOI:** 10.1186/1476-069X-13-41

**Published:** 2014-05-28

**Authors:** George D Bittner, Chun Z Yang, Matthew A Stoner

**Affiliations:** 1 CertiChem, Inc., 11212 Metric Blvd, Suite 500, Austin, TX, USA; 2 Department of Neuroscience, The University of Texas, Austin, TX, USA

**Keywords:** BG1Luc, Bisphenol A, BPA, Estrogenic activity, MCF-7, BG1Luc, Polycarbonate plastic, Human health

## Abstract

**Background:**

Xenobiotic chemicals with estrogenic activity (EA), such as bisphenol A (BPA), have been reported to have potential adverse health effects in mammals, including humans, especially in fetal and infant stages. Concerns about safety have caused many manufacturers to use alternatives to polycarbonate (PC) resins to make hard and clear, reusable, plastic products that do not leach BPA. However, no study has focused on whether such BPA-free PC-replacement products, chosen for their perceived higher safety, especially for babies, also release other chemicals that have EA.

**Methods:**

We used two, well-established, mammalian cell-based, assays (MCF-7 and BG1Luc) to assess the EA of chemicals that leached into over 1000 saline or ethanol extracts of 50 unstressed or stressed (autoclaving, microwaving, and UV radiation) BPA-free PC-replacement products. An EA antagonist, ICI 182,780, was used to confirm that agonist activity in leachates was due to chemicals that activated the mammalian estrogen receptor.

**Results:**

Many unstressed and stressed, PC-replacement-products made from acrylic, polystyrene, polyethersulfone, and Tritan™ resins leached chemicals with EA, including products made for use by babies. Exposure to various forms of UV radiation often increased the leaching of chemicals with EA. In contrast, some BPA-free PC-replacement products made from glycol-modified polyethylene terephthalate or cyclic olefin polymer or co-polymer resins did not release chemicals with detectable EA under any conditions tested.

**Conclusions:**

This hazard assessment survey showed that many BPA-free PC- replacement products still leached chemicals having significant levels of EA, as did BPA-containing PC counterparts they were meant to replace. That is, BPA-free did not mean EA-free. However, this study also showed that some PC-replacement products did not leach chemicals having significant levels of EA. That is, EA-free PC-replacement products could be made in commercial quantities at prices that compete with PC-replacement products that were not BPA-free. Since plastic products often have advantages (price, weight, shatter-resistance, etc.) compared to other materials such as steel or glass, it is not necessary to forgo those advantages to avoid release into foodstuffs or the environment of chemicals having EA that may have potential adverse effects on our health or the health of future generations.

## Background

Plastic resins are made by polymerizing one or more monomers in the presence of additives such as antioxidants, initiators, catalysts, thermal stabilizers, etc. More chemicals such as other antioxidants, thermal stabilizers, plasticizers, impact modifiers, clarifiers, colorants, mold release agents and rheology modifiers are then often added to the resin that is then heated and molded in various stages to make a product, or a single piece of a multi-piece product, each often composed of different chemicals. The final plastic product may receive yet-more chemical additives such as inks. All such additives are typically not part of the more chemically-tightly-bound, polymerized-monomer backbone of the plastic product. Since polymerization is not complete, unpolymerized monomers such as bisphenol A (BPA) can leach from the finished product, as can any additive. Furthermore, the high heat and other stresses of the manufacturing processes often generate additional leachable “degradation chemicals” that might have EA [1-3].

Until recently, many reusable, hard and clear, plastic products were commonly synthesized using BPA, a chemical known to have estrogenic activity (EA) that leaches from polycarbonate (PC) plastic products [4-6]. PC plastics were recently not recommended for use as baby products by the US FDA and such use has also been limited or banned in some other jurisdictions such as Canada, Minnesota and some EU countries; PC plastics are now not as widely used as they were several years ago in many consumer products [6,7]. However, PC-replacement products made from other BPA-free polymers might also release monomers having EA. Such BPA-free polymers would include acrylic, cyclic olefin copolymer (COC), cyclic olefin polymer (COP), glycol-modified polyethylene terephthalate (PETG), polyether sulfone (PES), polystyrene (PS), Tritan™ copolyester [3] and BPS [7,8] resins. PC-replacement products made from such resins might also release other additives that exhibit EA used in their manufacture such as triphenyl phosphate (TPP) or degradation chemicals such as phenols [3,9].

Chemicals are said to have EA if they mimic *in vitro* and/or *in vivo* actions of naturally occurring estrogens such as 17β-estradiol (E2). EA is the most-studied form of such endocrine disruptor activity [6,10-12]. Chemicals with EA bind to one or more estrogen receptor (ER) subtypes [8,13,14], and can produce various adverse health effects in mammals, such as early menarche, reduced sperm counts and other altered functions of reproductive organs, obesity, and increased rates of some cancers [6,12,15]. Fetal, infant and juvenile mammals have been reported to be especially sensitive to low doses of chemicals that exhibit EA [4-6,12,15].

Given these considerations, we performed a hazard-analysis survey that examined the leaching of chemicals with EA in over 1000 assays from 50 reusable, hard and clear, PC-replacement products. We used two well established *in vitro* mammalian cell-based assays [16,17] validated by ICCVAM/NICEATM/OECD (BG1Luc assay) or that has been undergoing validation (MCF-7 assay: [18]). Leaching of more- or less-polar chemicals was assessed by extracting PC-replacement products with more-polar (water or saline) or less-polar (ethanol (EtOH)) solvents, or mixtures of water and EtOH. The effects of some aspects of common-use stresses was tested by exposing PC-replacement products to autoclaving, microwaving, or UV radiation prior to solvent extraction.

We report that chemicals with EA were released (leached) from many unstressed BPA-free PC-replacement products (including products for babies). Stressing these PC-replacement products, especially with various forms of UV radiation often increased the probability and/or level of release of chemicals with EA. However, most importantly, some reusable, BPA-free, hard and clear PC-replacement products could be found that did not release chemicals with EA, even after exposure to UV or other stresses. Consequently, it is possible to manufacture EA-free PC-replacement products in commercial quantities and thereby avoid potential adverse consequences to the environment or human health due to release of xenobiotic chemicals with EA [6,11,12,15,19,20].

## Methods

### Materials

#### Survey of plastic products

Fifty reusable PC-replacement products made from seven types of resins (acrylic, COC, COP, PES, PETG, PS, Tritan™) were obtained from 2010–2013 by purchase at retail outlets. These products included many items for which BPA-free resins are commonly used such as baby bottles, reusable water bottles, food storage, packaging, cups, medical supplies, and labware. These items were branded by well-known firms such as Camelbak, Nalgene, Dr. Weil, Born Free, AVENT, Costar, Crate and Barrel, Green-to-Grow, and LocknLock. There was no consistent price differential for products made with different resins. We tested each product with various (not necessarily the same) combinations of extraction solvents and stresses using two different assays, MCF-7 and BG1Luc (see below). We often stopped testing a product if a particular type of extraction solvent or stress showed that the product leached chemicals having significant levels of EA. That is, the aim of this study was not to perform an exhaustively-complete study of responses to all possible stresses and extraction conditions for a few products, but rather to survey a larger sample of PC-replacement products and assess whether some released chemicals with EA whereas others were potentially EA-free.

#### MCF-7 and BG1Luc cells

MCF-7:WS8 (MCF-7) cells were a gift from Dr. V. Craig Jordan, then at Northwestern Medical School, now at Georgetown Medical School. Every 2–3 months the MCF-7 cells were replaced with stocks from the same MCF-7 primary source to maintain more uniform MCF-7 cell characteristics throughout the study. BG1Luc cells were licensed from the University of California, Davis. Media and medium supplements (RPMI (Roswell Park Memorial Institute)-1640 Medium, DMEM, FBS, nonessential amino acids, *l*-glutamine, penicillin, streptomycin) used to initially grow and maintain the MCF-7 and BG1Luc cells were purchased from Invitrogen (Grand Island, NY, USA). Insulin was purchased from Sigma (St. Louis, MO, USA).

MCF-7 and BG1Luc cells were maintained in polystyrene T-75 flasks (BD Falcon, BD Biosciences, San Jose, CA, cat#353136) and polystyrene T-25 flasks (CytoOne, USA Scientific, Ocala, FL, cat#CC7682-4825). MCF-7 cells were seeded into 96-well flat bottom PS polystyrene plates (BD Falcon, cat#353075) and BG1Luc cells were seeded into 96-well white wall/clear bottom plates (Greiner Bio-One, Monroe, NC, cat#655098). Ethanol was obtained as 100% from various sources (OmniPur, EMD-Millipore, Billerica, MA; Acros Organics/Fisher Scientific, Pittsburgh, PA or Sigma-Aldrich, St. Louis, MO). Water was distilled on-site in an all-glass system and collected directly into glass before use in extractions. Extractions were performed in borosilicate glass tubes.

### Equipment

We used Labconco Class II Biosafety Hoods (Kansas City, MO, USA) equipped with a 254 nm fluorescent bulb to enclose EpMotion 5070 robotic workstations (Eppendorf, Hamburg, Germany) for serial dilutions of test chemicals, cell seeding, and media changes in 96-well plates [3]. A Tristar Luminometer (Brethold Technology, Germany) was used to measure luminescence in BG1Luc assays. A Bio-Tek PowerWavex and a Bio-Rad 96-well plate reader spectrophotometer were used to measure DNA content in MCF-7 assays, as previously described [3,18].

### Protocols to stress plastic products

There are no regulatory protocols for stressing plastic products to test for leaching of chemicals with hormonal activity. Hence, we devised microwave, autoclave, and UV stresses described below to simulate various aspects of the short- or long-term effects of such common-use stresses of microwaving, boiling (moist high heat) or germicidal or UV exposure experienced by various types of PC plastics such as food containers, water bottles, or baby bottles. Some of these stresses have been previously described [3].

For most microwave stresses, 4x4 mm square pieces of plastic were placed into glass beakers in a 1200 W microwave oven set on “high” for two minutes, and then allowed to rest for 30minutes. The cycle was repeated 10 times. Some samples were placed in EA-free polypropylene (PP) tubes, and then microwaved on “high” setting for three minutes with a resting time 30minutes between stresses. The cycle was repeated 5 times. We did not detect consistent differences in leaching after the two protocols.

For autoclave stresses, plastic products were enclosed in individually crimped packets of EA-free aluminum foil and placed in a Tuttnauer autoclave at 134°C for 8 minutes.

For UV stresses, the UV radiation in sunlight is often classified [21] as UVC (100–280 nm), UVB (280–315 nm), and UVA (315–400 nm); visible wavelengths are from 400–750 nm. Most UVC wavelengths are filtered by the ozone layer before reaching the earth’s surface. However, UVC wavelengths are used in some germicidal UV devices, e.g., to sterilize baby bottles. For UV stresses, the protocols were as follows:

(1) Long wavelength (315–400 nm) UVA stresses that simulate many aspects of UV in sunlight: Samples were placed in a Q-Lab QUV unit containing UVA-340 nm bulbs to simulate exposure to moisture-free sunlight between 295 nm and 365 nm for 80 hours at 45–50°C.

(2) Short wavelength (100–280 nm) UVC stresses that simulate many aspects of UV in germicidal sterilizers: Samples were placed on aluminum foil in a Labconco Biosafety hood about 24” from a germicidal fluorescent light (maximum intensity wavelength of 254 nm) for 24 hours.

### Extracts of plastic products

Two to five grams of unstressed or stressed samples of PC-replacement products were added to sterile glass test tubes. The tubes were placed under a germicidal UV light for 30 minutes to sterilize the samples before adding an extraction solvent to produce a final concentration of 1.0 g/mL. Such brief UVC exposures do not alter leaching [3]. The extraction solvents consisted of saline-based solution (saline: RPMI-1640 Medium without phenol red), 100% EtOH, 10-50% aqueous EtOH, or distilled water. Most samples were extracted at 40°C for 240 hours in an incubator shaker. Saline extracts were diluted 2× with 2× estrogen-free medium (EFM) in a 1:1 ratio and then further diluted 1-4× with EFM so that the highest starting product concentration applied to wells was 0.125-0.500 g product/mL. [EFM was modified from cell maintenance media (see below) by replacing 10% FBS with 1% charcoal-stripped FBS and 4% charcoal-stripped calf serum and phenol red-free RPMI-1640.] EtOH extracts were concentrated 10× by evaporation and then diluted 100× with EFM to produce a highest starting concentration of 0.1 g product/mL to be applied to wells.

### MCF-7 assays

An MCF-7 cell line was used in a robotized version of the MCF-7 cell proliferation assay [3,18] that has been employed for decades in manual format to reliably assess EA [16,22]. The assay is currently undergoing validation for international use by ICCVAM/NICEATM [18]. Chemicals with EA bind to ERs and activate the transcription of estrogen-responsive genes, which leads to proliferation of MCF-7 cells.

As previously described in detail [3,18], cell maintenance media was used to grow and maintain the MCF-7 cells. This media consisted of RPMI-1640 media with non-essential amino acids, 10 μg/mL phenol red, 4 mM *l*-glutamine, 6 ng/mL insulin, 100 units/mL penicillin, 100 μg/mL streptomycin, and 10% fetal bovine serum (FBS). EA assays were performed in EFM. Each test extract at each concentration was added in triplicate or quadruplicate to 96-well plates containing MCF-7 cells in EFM. After six days exposure to test chemicals or extracts, the cell culture medium was aspirated and the amount of DNA/well, an indication of cell numbers, was assayed using a microplate modification of the diphenylamine assay [3,18].

### BG1Luc assays

The BG1Luc4E2 cell line (aka BG1Luc) responds to estrogenic chemicals with the induction of firefly luciferase [17]. The BG1Luc assay has been approved as a screening method for estrogenic chemicals by OECD, EPA, and ICCVAM/NICEATM [23]. BG1Luc cells were maintained in cell culture medium that consisted of phenol red-free DMEM with 8% FBS, 100 units/mL penicillin, and 100 μg/mL streptomycin, L-glutamine and sodium pyruvate. Prior to assaying for EA, BG1Luc cells were placed for 3 days in EFM that was modified from cell culture medium by replacing 8% FBS with 4.5% charcoal-stripped FBS and substituting phenol red-free DMEM for phenol red-free containing DMEM. Acclimated cells were then seeded at 10,000 cells per well in 100 μL EFM in 96-well plates for 24 hours, followed by a 24 ± 6 hours incubation with test extracts in triplicate.

Cytotoxicity was assessed as described by ICCVAM/NICEATM [23] and Yang et al. [18]:

Cells were visually observed under an inverted light microscope immediately before terminating incubation. Cellular cytotoxicity was assessed using scoring parameters 1 = normal cell morphology, 2 = low cytotoxicity (10 - 50% of cells with altered morphology), 3 = moderate cytotoxicity (50- 90% of cells had altered morphology), and 4 = high cytotoxicity (few or no cells visible). Test substance concentrations with a cytotoxicity score of 2 or higher were excluded from further analyses.

Cell culture medium was aspirated, cells were lysed, and luciferase was measured in an automated microplate luminometer (Tristar Berthold Technology, Germany) with the Promega Luciferase Assay System (Promega, Madison, WI, USA) following the manufacturer’s protocol.

### Calculation of EA

The estrogenic effect of a test chemical or extract on cell proliferation or luciferase activity was calculated as %RME2, a percentage of the maximum DNA/well (or the Relative Luminescence Unit, RLU) produced by the maximum response relative to 17β-estradiol (E2, positive control). This value was corrected for the background (DNA or RLU in MCF-7 or BG1Luc, respectively) response to the vehicle (negative) control. We incorporated both a vehicle control (VC) and “sham” control (SC) in each experiment. The VC was the vehicle used for that particular assay. The SC was the vehicle taken through all steps that were used to assay the test sample/test extract. For both SCs and test extracts, %RME2 was calculated by subtraction of the VC value from the SC or test sample value, followed by normalization of each adjusted value to the maximum E2 value measured in the experiment (set at 100%) and the VC (set at 0%). Typical value of an SC was 0% ± 10% RME2. However, if the EA of an SC were greater than 15% RME2, then the entire experiment was rejected.

The EA of a test chemical or extract was classified as detectable if the EA effect was greater than 15%RME2, which was greater than three standard deviations (SD) of the SC response for that experiment. Therefore, 15%RME2 is a conservative measure of EA detectability. Stimulation of MCF-7 proliferation or BG1Luc Luciferase expression by test chemicals or extracts was confirmed as estrogenic (rather than non-specific effects) by suppression of the EA by co-incubation with the anti-estrogen (ICI 182,780 (ICI) at 10^-7^ - 10^-8^ M.

We saw no examples of an unsuppressed agonist response using either assay. That is, these *in vitro* assays rarely produce false positive responses [3,18,24]. Note also that we have limited our analyses to whether the plastic or chemical exhibited EA that was statistically significantly (p < 0.01) greater than any EA detected in VC or SC samples. We have not attempted to statistically compare absolute %RME2 values for different extracts or plastic products because extraction procedures, while similar, were not exactly the same for the two cell lines and the chemicals, much less their concentrations, in extracts were not known.

## Results

### MCF-7 and BG1Luc assays of PC-replacement products with EA

**Figure 1:**
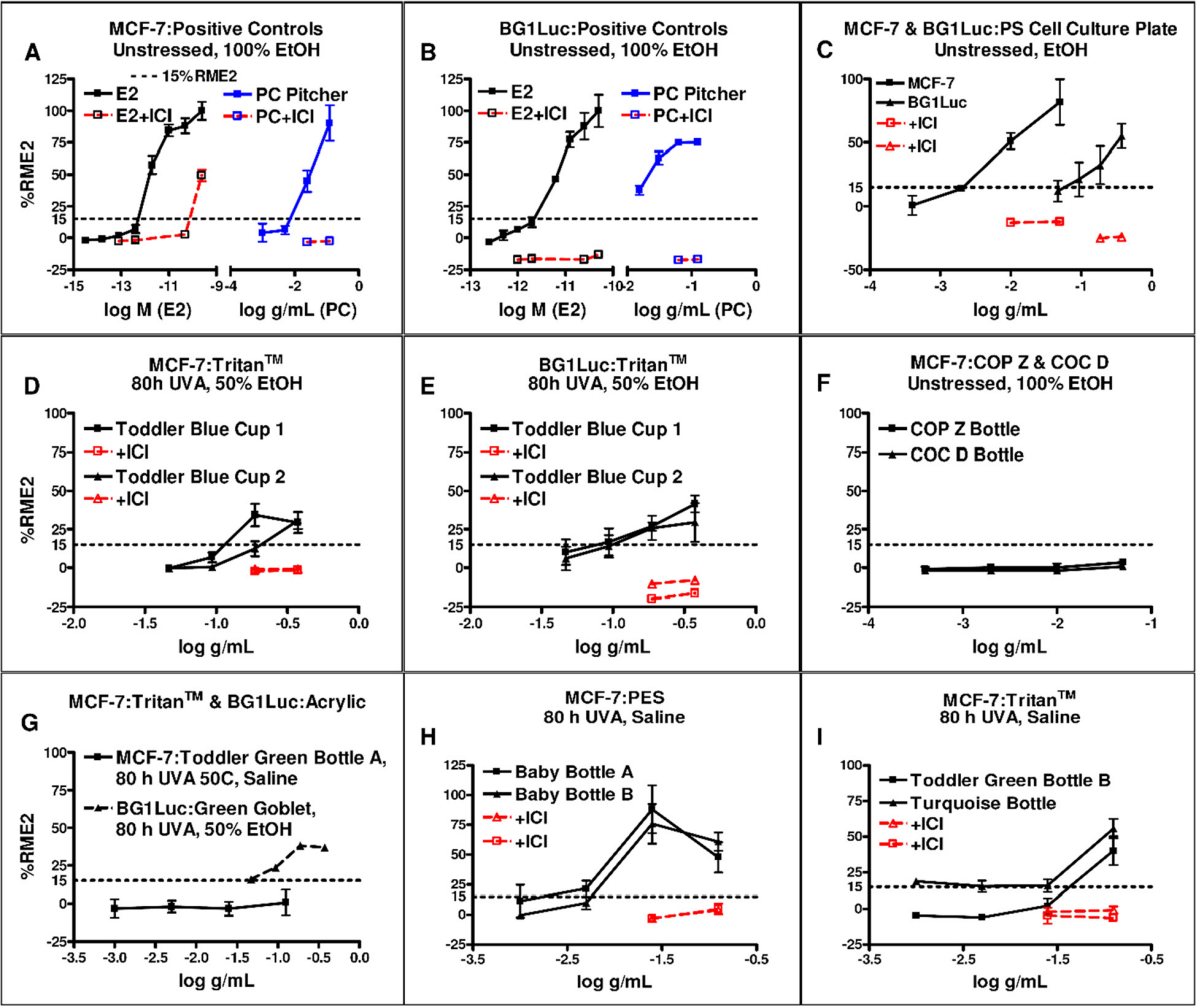
**Concentration-response curves for E2 and plastic extracts.** Mean and SD data for concentration (g/mL or M) – response (%RME2) curves of luciferase activity in BG1Luc cells and MCF-7 cell proliferation by extracts of PC **(A,B)** or PC-replacement **(C-I)** products. Black lines and associated data points show agonist activity for all data points not associated with toxicity (see Methods). Red lines and associated data points show results of exposure of extracts to 10^-8^ M ICI. Horizontal dotted lines show 15%RME2 values that are significantly (p < 0.01) greater than the vehicle (VC) and sham (SC) controls. PC-replacement products. Panel **C**. Costar 3300 tissue culture plate. **D**. Cup.1: Camelbak kids reusable water bottle, blue; Cup 2: Nalgene kids reusable water bottle, blue. **E**. Cup.1: Camelbak kids reusable water bottle, blue; Cup 2: Nalgene kids reusable water bottle, blue. **F**. Zeonor COP bottle. Topas COC bottle. **G**. AVENT baby bottle, green; Crate and Barrel wine glass, green. **H**. Born Free baby bottle A; Green to Grow baby bottle B. **I**. Dr. Weil baby bottle; Contigo reusable water bottle, turquoise.

**Figure 2:**
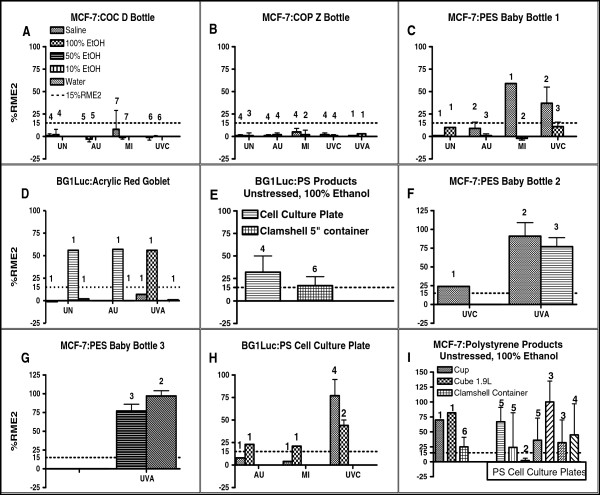
**EA of PC-replacement products assessed by MCF-7 or BG1Luc assays.** Means of highest value and SD of %RME2 from dose–response curves of various extracts of acrylic, COC, COP, PES, PS PC-replacement products as stated in key to each panel. Number of independent assays for each type of product extract (e.g., EtOH concentration, saline) given above the bar graph. Each assay consisted of three replicates for that extract whose SD was typically very small (as indicated in Figure 1). Unstressed = UN, AU = autoclave stress, MI = microwave stress, UVA and UVC = long and short wavelength UV stresses, respectively. Each assay for each product is considered by itself whether its EA is significantly greater than its VC or SC EA, i.e. > 15%RME2 (see Methods). We do not statistically compare EA values obtained for different plastic products because extraction procedures and concentrations, while similar, are not exactly the same. PC-replacement products. Panel **A**. COC Topas bottle. **B**. Zeonor bottle. **C.** Green to Grow baby bottle. **D**. Crate and Barrel wine glass, red. **E**. Costar 3585 cell culture plate; Dart clamshell container. **F**. Born Free baby bottle. **G**. Avent baby bottle. **H**. Costar tissue culture plate. **I.** Chinet cut crystal cup; Click Clack Cube; Dart clamshell container.

**Figure 3:**
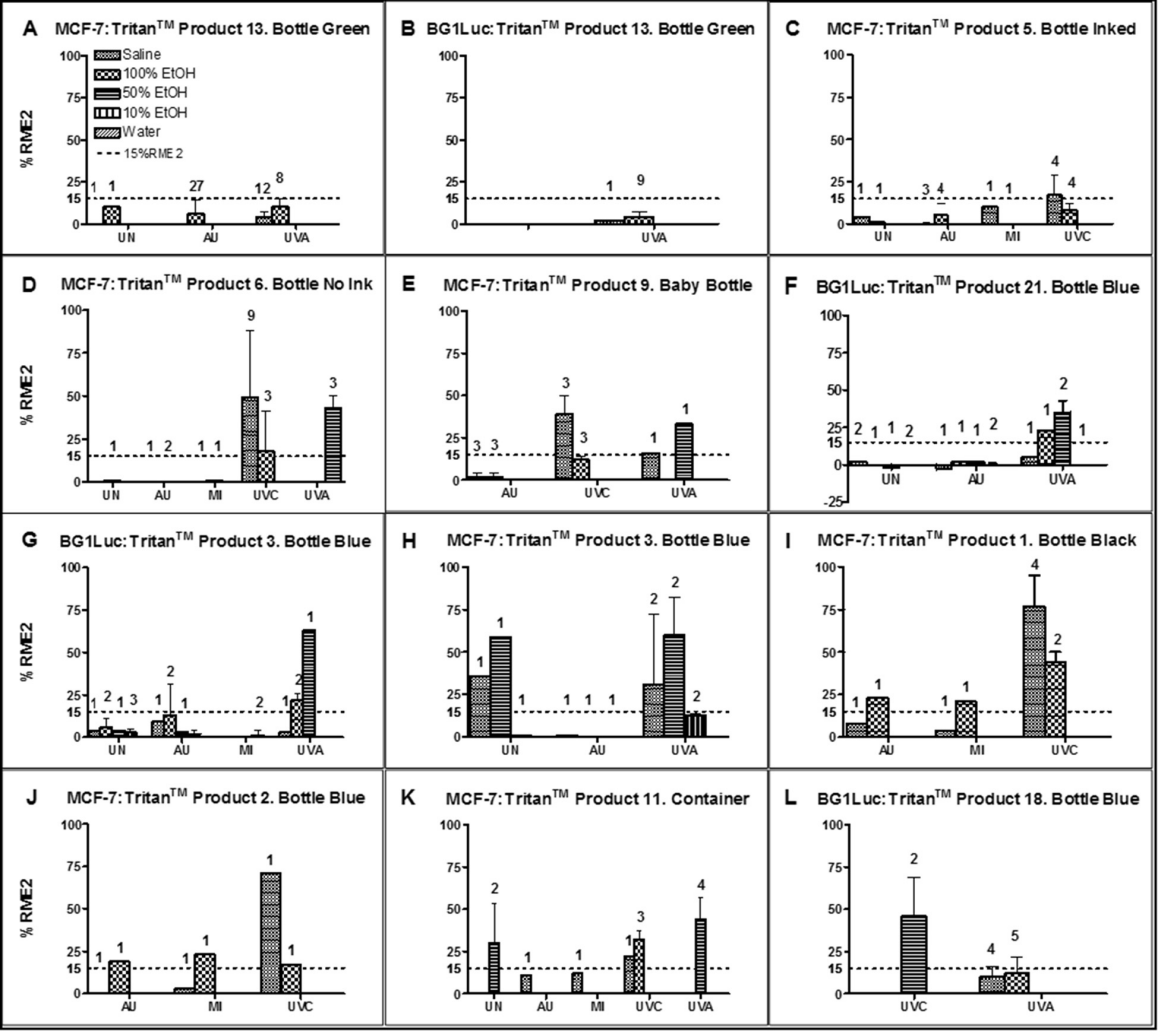
**EA of PC-replacement products assessed by MCF-7 or BG1Luc assays.** Means of highest value and SDs of %RME2 for sets of dose–response curves for Tritan™ products. Abbreviations as described for Figure 2. PC-replacement products. Panel: **A**. Nalgene reusable water bottle, green. **B**. Nalgene reusable water bottle, green **C**. Dr. Weil kids reusable water bottle. **D**. Dr. Weil baby bottle **E**. Evenflo baby bottle, clear. **F**. Nalgene reusable water bottle, blue. **G**. Camelbak kids reusable water bottle, blue. **H**. Camelbak kids reusable water bottle, blue. **I**. Camelbak reusable water bottle, black. **J.** Camelbak reusable water bottle, blue. **K**. Lock and Lock food storage container. **L**. Nalgene reusable water bottle, blue.

Our assays quantified the total EA of chemicals that leached from a plastic product, but could not determine the identity of those chemicals. The total EA of chemicals extracted from 50 BPA-free PC-replacement products was calculated as %RME2 from concentration (dilution)-response curves for MCF-7 and BG1Luc assays. Concentration-dependent increases in EA for E2 (in M) were the positive controls for MCF-7 assays (Figure 1A) and BG1Luc assays (Figure 1B). Concentration units for extracts of unstressed or stressed plastic products were given in grams of product/mL (Figure 1A,B). The statistically significant reduction of %RME2 by ICI (dashed lines, open symbols in Figure 1A,B) confirmed that any agonist activity was due to ER activation of EA, rather than a non-specific response. Extracts of PC products (Figure 1A,B) always exhibited the presence of chemicals having EA, almost-certainly mostly due to leaching of BPA [4-6]. Such EA was greatly reduced by ICI (Figure 1A,B).EA-positive concentration-response curves were observed for many PC-replacement products assayed in this survey. As representative examples from 50 products tested in over 1000 assays, Figure 1D and E show that both MCF-7 and BG1Luc assays detected chemicals with EA leaching from an unstressed toddler blue cup extracted using 50% EtOH. For this particular PC-replacement product, the MCF-7 assay was more sensitive in detecting EA than the BG1Luc assay. For some other products, the BG1Luc assay was more sensitive (also see Figures 2 and 3). However, the conclusion as to whether a PC-replacement product released chemicals with EA was almost always the same for the two cell lines (Figure 1C). Dose–response curves showing leaching of chemicals with EA were observed for both more-polar and less-polar extraction solutions, including 50% EtOH (Figure 1E) for many products, as did saline extracts for many products (also see Figures 2 and 3).Some extracts of PC-replacement products did not exhibit leaching of chemicals with EA. Figure 1F shows a dose–response curve for a 100% EtOH extract of unstressed COC and COP bottles that exhibited no detectable EA. Figure 1G shows a dose–response curve for a saline extract of a UVA-stressed green Tritan™ bottle with no detectable EA and a green acrylic wine goblet whose extract had EA. Colorless Tritan™ bottles or Tritan™ bottles of colors other than green often exhibited leaching of chemicals with EA (Figures 1I and 3).

### Survey of EA in leachates of BPA-free PC-replacement products

As shown in Tables 1 and 2 and Figures 2 and 3, we examined whether chemicals with EA leached into more-polar (saline) or less-polar (EtOH) extraction solvents from 50 unstressed and/or stressed reusable PC-replacement products. Stresses included autoclaving, microwaving, and/or UV irradiation. As previously reported [3], for some products EA positive responses were found in polar solvents, but not in non-polar or, alternatively, in non-polar but not in polar solvents. An unstressed or stressed product was classified as leaching chemicals with EA if statistically significant (p < 0.01) amounts of EA > 15%RME2 (see Methods) were detected in any extraction solvent for either MCF-7 or BG1Luc assays. Figures 2 and 3 show bar-graphed examples of the maximal EA (in %RME2) obtained from over 1000 concentration-response curves for various product types. Not all extracts or stresses were applied to each of 50 products tested in this survey in which we often stopped testing a product once a particular protocol showed that chemicals with EA leached from the product.

**Table 1:** Frequency of EA Leaching from UV-stressed PC-replacement products

**Ratio of EA**^**+ **^**products/total products tested for UV stresses for each type of resin**										
		**MCF-7 assays**				**BG1Luc assays**				**Either**
**Product type**	**n**	**Saline & water**	**100% EtOH**	**10% &50% EtOH**	**EA**^**+**^**/NT**	**Saline & water**	**100% EtOH**	**10& &50% EtOH**	**EA**^**+**^**/NT**	**Overall**
**Acrylic**	**3**	0/3	2/3	1/1	2/3	0/3	1/1	1/2	2/3	2/3
**COC**	**4**	0/2	0/2		0/2					0/2
**COP**	**1**	0/2	0/2		0/1					0/1
**PES**	**3**	4/4		2/2	3/3					3/3
**PETG**	**3**	1/3	0/2		1/3					1/3
**PS**	**9**	0/3	2/2							2/3
**Tritan™**	**25**	16/23	7/21	7/7	20/23	4/11	6/8	7/7	12/14	23/25

Relative number of UV-stressed (GUV or QUV) products exhibiting significant (p < 0.01) EA reported by resin type versus extract solvents for each assay.

“n” is the number of products of a resin that received at least one assay for UV-stress.

“blank cell” indicates that no UV tests done for that combination of conditions’.

“EA^+^/n Total” gives the number of products for an assay type showing significant leaching of chemicals having EA (EA^+^) with respect to the total number of products of that resin type (n).

“EA^+^/n Overall” gives the number of products showing significant leaching of chemicals having EA (EA^+^) with respect to the total number of products of that resin type (n) using *either* assay type.

All combinations of product, assay, and solvent types were not used, sometimes because unstressed products of a resin type exhibited significant EA (i.e., 9/11 *unstressed* products made from acrylic resins exhibited EA). For some cells that combine data for two extraction solvents (water and saline, 10% & 50% EtOH), the number of possible assays was double the number of products.

**Table 2:** Frequency of EA leaching from PC-replacement products

**Ratio of EA + products/total tested for each stress**						
**Product type**	**n**	**UN**	**MI**	**AU**	**UV**	**Overall**
**Acrylic**	3	2/3		2/3	2/3	2/3
**COC**	4	0/3	0/1	0/2	0/2	0/2
**COP**	1	0/1	0/1	0/1	0/1	0/1
**PES**	3	0/1	1/1		3/3	3/3
**PETG**	3		0/3	0/3	1/3	1/3
**PS**	11	9/11		0/1	2/3	9/9
**Tritan™**	25	2/6	3/10	3/14	23/25	23/25
**Total**	50	13/25	4/16	5/24	31/40	38/46

Relative number of products made from a resin type exhibiting significant (p < 0.01) EA for a given stress or for any stress.

Row labeled “overall” gives the number of products exhibiting EA for those products having at least three assays for at least 3 stresses (row labeled “Overall”).

Row labeled “Total” sums the data for that column.

n: number of products tested for any stress condition, blank column: no tests made for that particular combination of product and stress, UN: unstressed, MI: microwaved, AU: autoclaved, UV: UVA or UVC stress.

Unstressed extracts of some COC (Figure 2A), COP (Figure 2B), PES (Figure 2F), and Tritan™ (Figure 3A,C-G) products had no detectable EA in MCF-7 (Figure 3A, C-E) or BG1Luc (Figure 3F,G) assays. A few PC-replacement products, such as a COC bottle (Figure 2A), a COP bottle (Figure 2B), and a green Tritan™ bottle (Figure 3B) exhibited no detectable EA after one or more stresses, including one or more UV stresses, in more-polar or less-polar solvents. In contrast, one or more extracts of most PS, PES, and Tritan™ products (Figures 2C-I; 3C-L) exhibited EA in more-polar or less-polar solvents, especially when the product was exposed to UVA or UVC radiation. In general, Tritan™ products stressed with UVA or UVC radiation more frequently released chemicals with statistically significant levels (p < 0.01) of EA in both MCF-7 and BG1Luc assays compared to unstressed Tritan™ products. However, stressing *per se* did not *necessarily* increase the release of chemicals with EA. For example, compared to unstressed or UV-stressed products, extracts of autoclaved (AU) - or microwaved (MI) PC-replacement products often had lower EA in BG1Luc and MCF-7 assays (Figure 3C,D,G-K).

Although the analyses of the same extracts using the BG1Luc and MCF-7 assays produced slightly different %RME2 values (Figure 3A vs. B; G vs. H), the results of both assays almost-always led to the same conclusion that a given product did (Figure 3G,H) or did not (Figure 3A,B) release chemicals with detectable EA. Detection of EA in some but not all extracts of different products made from the same type of resin was not surprising, and likely resulted from different additives and impurities (catalyst residues, thermal degradation products, etc.) in the different resins or products and/or differences in their processing [3], as well as differences in the highest concentration tested (see Methods). Similarly, obtaining different %RME2 EA values of the same product for different extract solutions using the same assay (Figures 2, 3) was not surprising because chemicals with EA can be more-polar or less-polar and are more efficiently extracted with a solvent that is more-compatible with their polarity. The relative ability of more-or less-polar solvents to extract and detect chemicals with EA from UV-stressed products is summarized in Table 1. Water or saline extracts or water/EtOH mixtures were more effective than 100% EtOH in detecting EA leaching from Tritan™ type products when either MCF-7 or BG-1Luc assays were used. Conversely, for acrylic-based resins, 100% EtOH as a solvent was more effective than water-based solvents in extracting chemicals with EA.

Table 2 summarizes the findings for total EA in the leachates of the 50 products sorted according to the 7 types of resins used to manufacture the product. The data summarized in Tables 1 and 2 and Figures 1, 2, and 3 demonstrate that more than one type of extract and type of stress are needed to detect whether a given product will leach chemicals having detectable EA. Considering all assays of unstressed products for all resin types, 13/25 released chemicals with statistically significant (p < 0.01) levels of EA. Following microwave stress, only 4/16 of all products exhibited release of chemicals with EA and only 5/24 exhibited EA after autoclave stress. That is, some stresses might have reduced the release of chemicals having EA. Autoclaving, for example, might extract chemicals having EA so that after autoclaving, the stressed product subsequently releases chemicals having less total EA. In contrast, following UV stresses (UVA and/or UVC), 32/41 products demonstrated leaching of chemicals with EA, including most acrylic, PES, PS, and Tritan™ products. That is, UV stresses increased the probability (and perhaps the levels) of the total EA of chemicals leaching from PC-replacement products.

For PC-replacement products that received at least 3 assays of different types of extraction solvents or stresses (Table 2), 38/46 exhibited significant release of chemicals with EA in at least one extract type, including most products made from (2/3) acrylic, (3/3) PES, (9/9) PS, and (23/25) Tritan™ resins. That is, UV stresses significantly (p < 0.03, Chi Square Test) increased the probability that a product would release chemicals with EA (38/46) compared to unstressed products (13/25). However, our data also showed that hard and clear, reusable, PC-replacement products that did not release any detectable EA for any type of stress or extract could be manufactured in commercial quantities from some COC, COP, and PETG resins.

### Effects of UV radiation on EA release from BPA-free PC-replacement products

The results described above indicated that exposure to UV radiation could increase the EA levels of extracts from most PC-replacement products assayed in this study. However, the depth of UV wavelength penetration into the product depends upon the structure of the polymer and its additives. Thus, even if chemicals with EA were produced by UV radiation in the presence of oxygen and such chemicals were readily extractable at the outer surface of a plastic container, the chemicals might not be produced and/or released from the inner surface of such containers.

**Figure 4:**
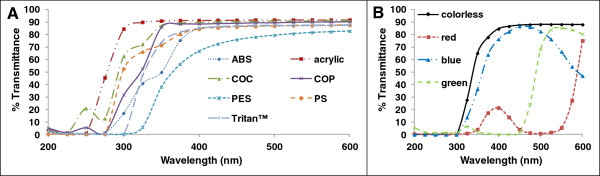
**UV transmission curves for some PC replacement products.** UV/Visible spectroscopy of PC-replacement products in Table 2 showing percent light transmittance at different wavelengths. **(A)** UV penetration for colorless bottles of resin type given in key. **(B)** UV penetration for colorless or colored bottles made from Tritan™ resin.

**Figure 5:**
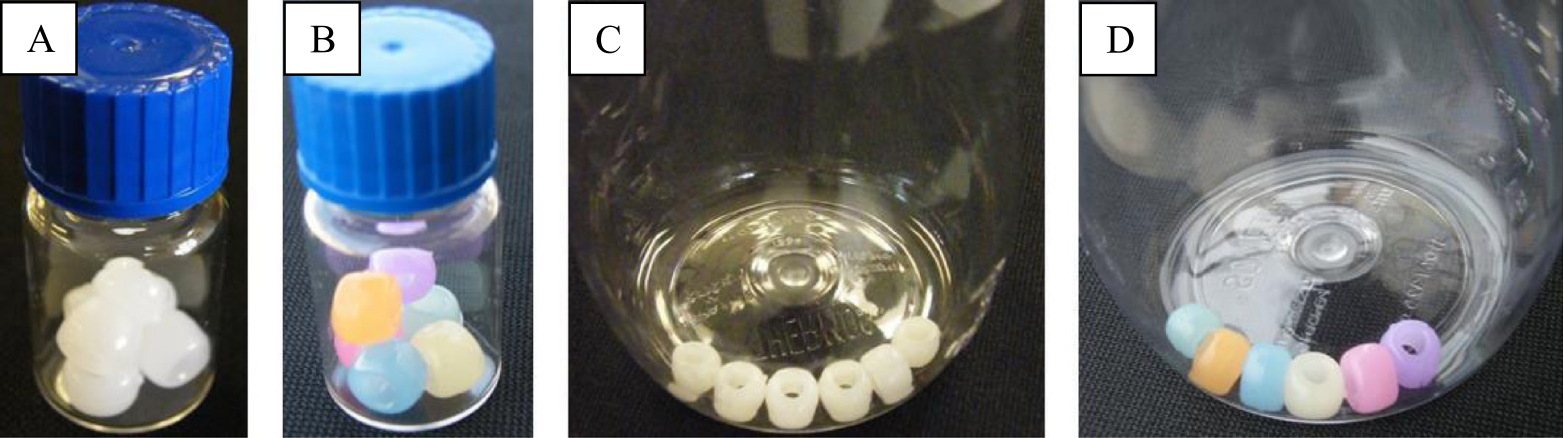
**UV light transmits to the interior of Tritan™ bottles.** UV-sensitive beads placed in colorless PC-replacement COC **(A,B)** or Tritan™ **(C,D)** bottles before **(A,C)** or after **(B,D)** a few seconds of outdoor exposure. Different beads change to a given color when exposed to UV, i.e., a color change, but not the specific color change, shows that UV light has been captured by the beads.

To examine this possibility, we determined the UV/Visible spectra in the range of 200–600 nm of blow-molded bottles of similar thickness made from various PC-replacement products (Table 3 and Figure 4). UV radiation is often classified by wavelength as UVA, UVB, and UVC ([21] See Methods). All products transmitted UV radiation over a range of wavelengths typical for each respective class of PC-replacement resins. Table 3 shows for colorless products that the PES bottle had the lowest percent penetration of UVA + B and UVA radiation. These data matched well with data shown in Figure 4A, in which PES had the lowest percent transmittance of the products tested in this study. The UV/Visible spectrum of acrylic indicated that it had the highest transparency of the products in this study, but had slightly lower percent transmittance of the UVA + B and UVA wavelengths than PS.The ability of UV light to penetrate these PC-replacement products was confirmed by inserting white UV-detecting beads that changed color when exposed to light in the range of 300–360 nm into bottles made from various resins. Different beads changed to different colors for the same UV exposure, i.e., the exact change color was not significant. Figure 5 shows white beads placed in the bottle prior to exposure to sunlight outdoors for a COP (Figure 5A) and for a Tritan™ (Figure 5C) bottle. Figure 5B and D shows that those beads inside either bottle changed color after a few seconds of outdoor exposure to UV in sunlight.

**Table 3:** Integrating radiometer data for HC bottles and containers

**Base resin**	**Product**	**Color**	**Thickness (mm)**	**UVA+B [J/cm**^**2**^**]**	**% UVA+B**	**UVA [J/cm**^**2**^**]**	**% UVA**
PES	Bottle	Colorless	1.30	1.00 [2.90]	34.4	0.98 [2.73]	36.0
ABS	Food storage	Colorless	1.88	1.04 [2.38]	43.9	1.08 [2.14]	50.5
PS	Food storage	Colorless	0.25	2.31 [2.82]	81.9	2.23 [2.72]	82.0
Acrylic	Goblet	Colorless	2.31	2.19 [3.02]	72.5	2.27 [2.85]	79.8
Tritan™	Bottle	colorless	1.70	1.56 [2.72]	57.3	1.78 [2.57]	69.0
	Toddler bottle	colorless	1.35	2.07 [2.78]	74.4	2.05 [2.88]	71.3
	Bottle	Green	1.62	0.03 [3.03]	1.1	0.020 [2.71]	0.70
	Bottle	Blue	1.62	0.90 [2.80]	32.1	0.88 [2.94]	29.9
	Bottle	Red	1.49	0.74 [2.68]	27.7	0.78 [2.47]	31.5

An integrating radiometer was used with two sensors to measure penetration of UVA + B and UVA radiation on August 7^th^ and 8^th^, 2013 in Austin, TX. The percentage of radiation that penetrated into the interior of the containers was calculated by integrating the energy over a ten minute interval in the sunlight without anything blocking the sensors (value inside of brackets), and then placed into the sealed containers for ten minutes. J = joules.

### Product color can significantly alter UV transmission

Table 3 and Figure 4B show that red, blue, and green colorants in Tritan™ bottles could reduce the percent transmittance relative to a colorless bottle. Figure 4B shows transmittance data for four different colored Tritan™ bottles of the same model, with walls 1.35 to 1.90 mm thick. The red bottle blocked most UV radiation 200–325 nm passing through the sample, with an increasing amount of penetration as the wavelength increases. The blue bottle blocked UV radiation below 325 nm, but allowed much UV radiation to pass at 325 - 400 nm. The green colored bottle showed the least penetration compared to the colorless or other colored bottles and blocked almost all (~99%) of the UVA and UVB radiation.

### Additives in BPA-free replacement products can exhibit EA

**Figure 6:**
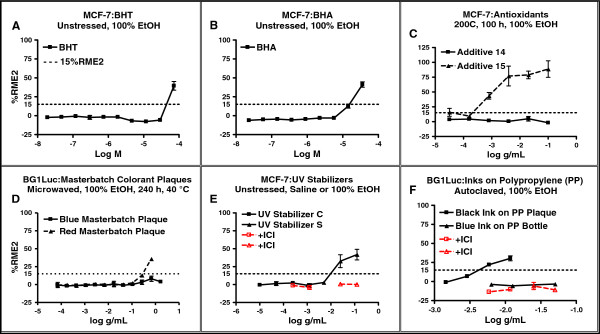
**Concentration-response curves for some common additives.** Dose–response curves for 6 additives used in the manufacture of various PC-replacement plastics. Key as for Figure 1. Panels **A-F**: antioxidants **(A-C)** tested as individual chemicals; colorants **(D)**, UV stabilizers **(E)** or inks **(F)** added to PP bottles or PP plaques. Without the additive, the PP plaques or bottles exhibited no detectable EA (data not shown).

In addition to polymerized monomers, PC-replacement products contain many other chemicals, including those added during resin or product manufacturing processes. Some of these additives might have EA and could potentially leach from the outer surface (e.g., during handling) or inner surface (e.g., ingestion of container contents). MCF-7 assays showed that two unstressed lower-molecular-weight antioxidant additives, butylated hydroxyanisole (BHA) and butylated hydroxytoluene (BHT), exhibited EA (Figure 6A,B). We tested other additives by adding them to PP resins that were formed into plaques or bottles that exhibited no detectable EA in the absence of any additional chemicals added during manufacture. When one commonly used higher-molecular weight antioxidant (processing stabilizer) was added prior the forming the PP resin into plaques (heat stress), the resulting plaques leached chemicals with EA. (Figure 6C). However, another higher-molecular-weight antioxidants did not exhibit EA even after many hours of heat stress (Figure 6C). Similarly, many plastic colorants contained in EA-free plaques added during manufacture exhibited EA, but others were EA-free after heat stress (Figure 6D). Our assays also showed that other additives such as EA-containing and EA-free UV stabilizers (Figure 6E) and inks tested on EA-free bottles or plaques (Figure 6F) were commercially available.

## Discussion

### Assessments of EA release from BPA-free PC-replacement products

While the release of various chemicals from plastic products had been well-studied [1,2], the specific leaching of chemicals with hormonal activity such as EA from many different types of unstressed or stressed plastics has only recently been examined [3]. BPA is by far the best studied as an estrogenic chemical released from reusable hard and clear PC-type products (for reviews see [4-6]). For example, Howdeshell et al. [25] reported that BPA with significantly detectable EA leaches from PC animal cages. Le et al. [26] reported that BPA leaches from PC drinking bottles. Yang et al. [3] reported that chemicals with EA leached from a small sample of commercially available PC products. However, prior to the current survey, there were few data on whether chemicals exhibiting EA leached from BPA-free PC-replacement products.

Our data from more than 1000 *in vitro* assays showed that many BPA-free PC-replacement products made from various types of resins, including PETG, PS, PES, and Tritan™ resins, could leach chemicals with EA, irrespective of whether these products were unstressed (13/25 in Table 2) or stressed (38/41 in Table 2) or which *in vitro* assay was used. Furthermore, agonist responses obtained using BG1Luc or MCF-7 assays were always inhibited by ICI, confirming that the observed agonist activity required ER activation. These data were consistent with a much smaller set of previous data using only MCF-7 assays showing that 14/28 unstressed products made from PS resins leached chemicals with EA (Table 1 of reference [3]) and that that four water bottles and a baby bottle made from Tritan™ resins (identified as PETG resins in Figure 3 of reference [3]) released chemicals with EA, especially when stressed with UVC (germicidal) radiation. Our much more extensive data in the survey reported herein also demonstrated that some unstressed and stressed colorless COC, COP, and PETG PC-replacement products (Tables 1, 2) did not release chemicals with detectable EA, indicating that it is possible to synthesize EA-free PC-replacement products for commercial use.

### UV stresses often increase leaching of chemicals with EA

While leachates obtained from unstressed products could contain chemicals with EA, exposure to UV radiation frequently increased the probability of leaching of chemicals with EA from those same products. For example, UV exposure produced release of chemicals with EA from all Tritan™ products, except for one green-colored bottle (Figures 1, 2, and 3). This increased probability of EA release could result from UV-dependent formation of new chemical (s) with EA and/or enhanced release of chemical (s) with EA that were already in the unstressed product.

It is not unexpected that UV exposure might cause chemical changes in a plastic. UVC wavelengths (100–280 nm) found in germicidal sterilizers are damaging to plastics; UVB (280–315 nm), and UVA (315–400 nm) [21] wavelengths in sunlight are also damaging to plastics, especially in the presence of oxygen at the inner and outer surfaces of a plastic product [27-33]. Although UVB wavelengths have greater energy and cause more UV degradation in plastics than UVA wavelengths, UVA wavelengths are more commonly found at the earth’s surface and can cause much degradation of PC-replacement polymers, especially with longer exposures [27,28,30,34]. In brief, if UV wavelengths associated with photo-oxidation are transmitted through the walls of a plastic product, significant degradation with subsequent leaching can occur on the non-exposed, inner, surface of the plastic [29].

Our UV transmission data (Figure 4, and 5, Table 3) demonstrated that UV wavelengths could completely penetrate to the inner surface of various PC-replacement bottles where chain scission or other chemical reactions could occur in the presence of oxygen, thereby potentially producing chemicals with EA that could leach into solutions contained within the product. Product manufacturers could attempt to minimize such UV penetration to the inner wall of their products. For example, our data (Figure 4B) suggest that the potential UV degradation at the inner surface of some green bottles would be lower compared to the colorless and blue Tritan™ bottles. However, green colorants would not guarantee that a green product would not leach chemicals with EA. As one example, a UV-stressed green acrylic wine goblet (Figure 1G) leached chemicals with EA, as did other green bottles made of various resins (data not shown). Hence, rather than trying to prevent release by using certain green colorants, we believe that the best solution to potential leaching of UV-produced chemicals having EA would be to use PC-replacement resins and additives that remain EA-free upon exposure to UV radiation.

### Additives and monomers can exhibit EA or be EA-free

PC-replacement products are manufactured in ways that almost-always use additives in addition to the monomers polymerized to make the resin. Such additives can leach from the plastic product. Our data (Figure 6) showed that some commercially-available additives such as antioxidants, colorants, processing stabilizers and inks had EA, whereas others were free of detectable EA (EA-free). Our previously published study [13] reported that some monomers (COC, COP, some PETG] now used to manufacture PC-replacement products should be EA-free and some COC, COP, and PETG products were indeed EA-free in the present study. Use of chemicals (monomers or additives) that exhibit EA to produce a PC-replacement product would almost-certainly increase the probability that the product would leach chemicals having EA. As one possible example, TPP is an additive used in the synthesis of Tritan™ resins [35] and TPP exhibits EA [9]. Hence, some of the EA we detected in extracts of Tritan™ products could arise from leaching of TPP and/or its degradation chemicals. However, our data also suggest that EA-free additives can be found that could be used to manufacture BPA-free PC replacement products from resins that were also EA-free, Such a fortuitous circumstance might account for our data that some COC, COP, or PETG products were EA-free.

## Conclusions

The results of our MCF-7 and BG1Luc assays demonstrate that extracts of many unstressed and/or stressed BPA-free PC-replacement products, including most acrylic, PES, PS and Tritan™-based products, release chemicals that can activate ER-dependent cell signaling, i.e., exhibit EA. In this survey of PC-replacement products, we quantitatively measure maximum effects of total EA in leachates (%RME2) relative to the maximum effect of E2) using two sensitive assays and various extraction protocols. We recognize that such *in vitro* data can only describe the existence of a possible hazard for consumption of chemicals with EA leaching from plastic products, not what risk that consumption might have to human health. We cannot calculate that risk in part because we and other scientists do not know how much total EA from plastics and other sources that anyone is exposed to, how many chemicals have EA, their relative EA, their release when exposed to different solvents or stresses, their metabolic degradation products or half-lives *in vivo*, whether chemicals with EA from different sources are additive or synergistic, and the appropriate levels of EA in males *versus* females at different life stages.

However, whatever the potential risk, our data for some types (COC, COP, and PETG) of BPA-free PC-replacement products show it is possible to use resins and additives to manufacture such PC-replacement products, including water bottles and baby products, in commercial quantities that are also free of detectable EA. Such data are important because other studies have reported that chemicals with EA in mammals can produce various adverse health effects such as early menarche, reduced sperm counts and other altered functions of reproductive organs, obesity, and increased rates of some cancers; some of these effects are produced at very low doses in fetal, infant, and juveniles [4,6,15]. Various studies from other laboratories have also suggested that chemicals with EA can produce measurable changes in the health of various human populations, e.g., on the offspring of mothers given diethylstilbestrol [4-6,12,15]. Since plastic products have advantages (weight, cost, impact strength, energy footprint, etc.) in various combinations compared to other materials such as steel or glass, it is not necessary to forgo these advantages of plastic in order to avoid release of chemicals having EA into foodstuffs or the environment that may have potential adverse effects on our health or the health of future generations. That is, our data show that producing EA-free PC-replacement products is not an impossible or extraordinarily costly task.

## Abbreviations

BPA: Bisphenol A; BPS: Bisphenol S; CCi: CertiChem Inc.; COC: Cyclic olefin copolymer; COP: Cyclic olefin polymer; E2: 17β-estradiol; EA: Estrogenic activity; EA-free: No detectable estrogenic activity; EFM: EA-free medium; ER: Estrogen receptor; EtOH: Ethanol; ICCVAM: Interagency Coordinating Committee on the Validation of Alternative Methods; NICEATM: National Toxicology Program’s Interagency Center for the Evaluation of Alternative Toxicological Methods; NIEHS: National Institute of Environmental Health Sciences; NIH: National Institutes of Health; NSF: National Science Foundation; OECD: Organization for Economic Cooperation and Development; PETG: Glycol-modified polyethylene terephthalate; PES: Polyethersulfone; PP: Polypropylene; PPi: PlastiPure Inc.; PS: Polystyrene; RPMI: Roswell Park Memorial Institute; SC: Sham control; TPP: Triphenyl phosphate; UV: Ultraviolet; UVA: Long wavelength UV; UVC: Short wavelength UV; VC: Vehicle control.

## Competing interests

CZY is employed by CCi. CZY and GDB own stock in CertiChem (CCi) and PPi. GDB is consulting CEO of CCi. MAS was employed by CCi. This is a research study. No products in this paper are manufactured or sold by CCi or CCi and most data were obtained on NIH grants to assay such products. CCi has put its MCF-7 and BG1-Luc robotic assays in the public domain.

## Authors’ contributions

All authors participated in the design of the experiments. CZY was primarily responsible for MCF-7 assays and MAS for BG1Luc assays at CCi. GDB primarily and CZY and MAS secondarily were responsible for writing the manuscript. CZY and MAS were primarily responsible for analyzing and plotting data. All authors read and approved the final manuscript.
